# Are integrative systematic tools efficient toward unraveling species diversity with the genus *Jania* (Corallinaceae, Rhodophyta)?

**DOI:** 10.1111/jpy.70167

**Published:** 2026-04-29

**Authors:** Clio Maridakis, Viviana Peña Freire, Gilberto Marani, Maria Tarin Sancho, Line Le Gall, Florence Rousseau

**Affiliations:** ^1^ Équipe Exploration, Espèces et Évolution, Institut de Systématique, Évolution, Biodiversité, UMR 7205 ISYEB CNRS, MNHN, UPMC, EPHE Muséum National d'Histoire Naturelle (MNHN) Paris France; ^2^ BioCost Research Group, CICA – Centro Interdisciplinar de Química e Bioloxía, Department of Biology, Faculty of Sciences Universidade da Coruña A Coruña Spain

**Keywords:** biogeography, *Cheilosporum*, COI, coralline algae, cox1, *Haliptilon*, integrative taxonomy, molecular phylogeny, morphometry, *psb*A, species delimitation

## Abstract

The articulated genus *Jania* currently comprises 54 accepted species, making it the fourth most speciose genus among corallines, following *Lithophyllum*, *Amphiroa*, and *Lithothamnion*. Unlike these other genera, *Jania* is relatively easy to identify at a generic rank. However, morpho‐anatomical characters are insufficiently discriminant for species identification, making DNA sequences essential for reliable species delimitation. We evaluated species diversity within *Jania* using the most comprehensive sampling to date, spanning a broad geographic range with a focus on the Mediterranean and Caribbean regions. Our data set comprised 186 specimens from the National Herbarium of the Muséum national d'Histoire naturelle (PC), including four type specimens with the basionyms *Jania micrarthrodia*, *Corallina polydactyla*, *Corallina mauritiana*, and *Jania digitata*. We also incorporated publicly available sequences from GenBank and Barcode of Life Data System (BOLD) to delimit species and infer their phylogenetic relationships using the *psb*A and COI genes. Through an integrative taxonomic approach, combining morpho‐anatomical traits, molecular systematics, and biogeography, we delineated 39 putative species—28% fewer than the currently accepted number. Most of the putative species had a restricted distribution, whereas five were widely distributed. We determined seven species from the European Atlantic and Mediterranean seas and six from the Caribbean. Furthermore, we demonstrated that intergenicular morphometry is an unreliable trait for species identification, highlighting the morphological plasticity of *Jania*. Many putative species remained unidentified (67%), while some of those putative species included specimens with different identifications. Additional sequences of type specimens would be crucial for further resolving taxonomy and bridging the gap between type‐bearing name and putative species delineated based on molecular data.

**Abbreviations**
ASAP: Assemble Species by Automatic Partitioning
BOLD: Barcode of Life Data System
CCA: canonical correspondence analysis
COI: cytochrome c oxidase I
DA: discriminant analysis
MCMC: Markov chain Monte Carlo
ML: maximum likelihood
MOTU: molecular operational taxonomic unit
mPTP: multirate Poisson Tree Process
PCA: principal component analysis
PCR: polymerase chain reaction
*psb*A: photosystem II protein D1
PTP: Poisson Tree Process
sPTP: single Poisson Tree Process
SSH: secondary hypotheses on species
SST: sea surface temperature

## INTRODUCTION

The genus *Jania* (Corallinales, Rhodophyta) is the fourth most speciose genus of coralline algae after *Lithophyllum*, *Amphiroa*, and *Lithothamnion* and currently comprises 54 accepted species (Guiry & Guiry, 2026). Species of *Jania* are distributed worldwide except in high latitudes (Guiry & Guiry, 2026) and are commonly observed in the shallow water of wave‐exposed shores (Johansen, 1976).

Among articulated corallines, the genus *Jania* was initially described based on morpho‐anatomical characters including the dichotomous branching pattern, terete to compressed intergenicula, and the presence of axial conceptacles (Irvine & Johansen, 1994; Johansen, 1969, 1981; Johansen & Womersley, 1994; Lamouroux, 1812). Johansen and Silva (1978) proposed to accommodate *Jania*, together with the genera *Cheilosporum* and *Haliptilon*, into the tribe Janieae, based on reproductive characters that differed from the tribe Corallineae by a set of features: thick, compact fusion cells bearing marginal carposporangial filaments; male conceptacles with narrow chambers and short canals; and a relatively small number of sporangia in each tetrasporangial conceptacle. Later, Kim et al. (2007) revised the generic delimitation and diversity of Janieae using 26 morpho‐anatomical characters and phylogenetic analyses of nuclear SSU gene sequences of 42 taxa. And following principle III of the current Madrid Code (Turland et al., 2025)—the principle of priority—the genera *Cheilosporum* and *Haliptilon* were synonymized under the older name *Jania*, rendering the genus monophyletic but increasing the variability of the diagnostic characters. However, the anatomical and morphological characteristics of *Haliptilon* and *Cheilosporum* are slightly different from *Jania* in branching structure (Kim et al., 2007; dichotomously branching and secondary often pinnate), in the shape of the intergenicula (terete to compressed, wedge‐shaped or distally lobed intergenicula), and on the conceptacle position. Moreover, *Haliptilon* differs by its pinnate thallus with axial intergenicula compressed and usually bearing subterete branchlets within which conceptacles can develop. *Cheilosporum* is defined by a subdichotomous thallus with intergenicula mostly compressed and bilobed and having conceptacles embedded within lobes. Phylogenies inferred from molecular characters revealed the weakness of these morpho‐anatomical characters to delimit those genera suggesting that they are prone to convergence phenomena.

Within *Jania*, taxonomy has also been challenging, as illustrated by the 72 names available for either infraspecific, synonymous, or uncertain taxa beyond the 54 currently accepted species names (Guiry & Guiry, 2026). Morphological identification has often been complicated by intraspecific variability due to phenotypic plasticity. For instance, Lugilde et al. (2019) relied on DNA sequences to identify specimens collected from maerl environments; these specimens exhibited an atypical morphology—characterized by an irregular pattern of dichotomies and secondary attachment discs—contrasting with the more robust intergenicula observed in samples from rocky habitats.

The lack of formal typification or sufficiently documented information on type specimens for many species also exacerbates the taxonomic confusion (Woelkerling, Harvey, & De Reviers, 2015a), hindering the reliable assignment of a species name to a specimen and the establishment of a consistent morpho‐anatomical species concept under a species name. The case of *Jania verrucosa* exemplifies these difficulties: This species has been variously treated as a valid species or as a heterotypic synonym of *J. crassa*. Due to the absence of a formal typification and the uncertainty surrounding the original material, which prevented the proposal of an unequivocal lectotype, Woelkerling, Harvey, and De Reviers (2015a) proposed rejecting the name *J. verrucosa* to mitigate nomenclatural instability. Consequently, the identity of specimens previously identified as *J. verrucosa* must now be reassessed to determine whether they belong to *J. crassa* (as confirmed for Australian specimens by Woelkerling, Harvey, & Reviers, 2015) or to another species. More recently, Nelson et al. (2023) revealed the identity of *J. crassa* by sequencing the isolectotype, and whereas the distribution of this species was considered widespread in New Zealand and Australia, the authors showed that the only specimen that matched the type sequence was from near the type locality in New Zealand, suggesting that this species has rather a limited distribution.

A revision of the genus *Jania* using molecular systematics and a comprehensive sampling is urgently needed to better assess species diversity and distribution. Over the past 2 decades, taxonomic studies on *Jania* have been limited either by their geographic span (mostly focusing on restricted regions) or by the use of morphological characters alone (Harvey et al., 2020; Lugilde et al., 2017; Mateo‐Cid et al., 2013; Wai, 2018). Molecular systematics studies have been conducted in Atlantic Spain (Lugilde et al., 2019), Brazil (Tâmega et al., 2021), New Zealand (Nelson et al., 2023; Twist et al., 2018), and southeastern Australia (Macagnan et al., 2023). A recent study by Macagnan et al. (2023) resulted in an overrepresentation of sequences from the temperate Australasian realm in GenBank, particularly for *J. crassa*, *J. pedunculata*, *J. micrarthrodia*, *J. sagittata*, and *J. rosea*. In these molecular studies, the *psb*A and COI genes were the markers most commonly used to delimit species boundaries and the most represented in molecular databases.

With the aim to advance our understanding of *Jania* species richness across a broad geographic range, here we present the most comprehensive sampling of *Jania* specimens to date, using an integrative approach that combined molecular data (*psb*A and COI gene sequences), ecological distribution data, thermal tolerance, and morphological variability assessed through morphometric analyses. In particular, we focused on the distribution of *Jania* species along the French European and Caribbean coasts, drawing on collections from recent campaigns organized by the Muséum national d'Histoire naturelle.

Furthermore, aligning with ongoing efforts in coralline algae to obtain DNA sequences from type specimens (Bustamante et al., 2019; Calderon et al., 2021; Gabrielson et al., 2018, 2023; Hernandez‐Kantun et al., 2015; Hind et al., 2014; Miller & Hughey, 2024; Peña, Bélanger, et al., 2021; Peña, Harvey, et al., 2021; Pezzolesi et al., 2019; Puckree‐Padua et al., 2022; Richards et al., 2017, 2021; Schipper et al., 2023; Wade et al., 2023), we successfully sequenced the *psb*A gene for four type specimens of *Jania* housed in the PC herbarium (herbarium acronyms follow Thiers, 2026, continuously updated). This study thus provides insights into the global diversity and systematics of the genus *Jania* and underscores the importance of integrative taxonomic approaches in clarifying species boundaries within this taxonomically challenging group.

## MATERIALS AND METHODS

### Field collections, DNA extraction, amplification, and sequencing

One hundred and eighty worldwide samples, recently collected and stored at the Paris cryptogamy collection (PC; see Table S1), were complemented with one specimen collected in the 1950s in Algeria by Jean Feldmann and four type specimens housed in PC for which SANGER sequencing was successful: *Jania micrarthrodia* (type locality: Australia, lectotype PC0028702), *Corallina polydactyla* (currently regarded as a synonym of *Haliptilon polydactylum*; type locality: Reunion island, lectotype PC0028626), *C. mauritiana* (currently regarded as a synonym of *H. mauritianum*; type locality: Mauritius, syntype PC0028656), and *J. digitata* (currently regarded as a synonym of *Arthrocardia palmata*; type locality: South Africa, isotype PC0028700). Tissues selected for DNA extraction were cleaned of epiphytes under a stereomicroscope. DNA extraction was performed using a NucleoSpin® 96 Tissue kit (Macherey‐Nagel, GmbH and Co. KG, Germany) or using the DNeasy 96 plant kit (Qiagen). The mitochondrial COI‐5P fragment was amplified using the primer pairs GazF1 and GazR1 (Saunders, 2005) or GazF1 and GCOR3 (Peña Freire et al., 2014). The *psb*A gene locus was amplified using *psb*A‐F1 and *psb*A‐R1 or *psb*A600R (Yoon et al., 2002) or *psb*A‐F1 and *psb*A‐R1bis, a newly developed primer (reverse, 5′ GCTAAATCTAGTGGGAAGTTATG 3′). The thermal profile for polymerase chain reaction (PCR) amplification for COI‐5P gene followed Saunders and McDevit (2012), and the thermal profile for PCR amplification for the *psb*A gene followed Bittner (2009). The PCR protocol for amplification of the extractions, followed the protocol published in Peña et al. (2015). For type specimens, DNA was separately extracted from the other specimens, in a clean room reserved for extraction and without any other extractions the same day. The extraction was done according to Nelson et al. (2023) and Nelson et al. (2025), using QIAamp®DNA Micro Kit (Qiagen S.A.S., Les Ulis, France) following the manufacturer's protocol. The *psb*A gene sequence of type material was obtained using the primer pair *psb*A‐F1 and *psb*A600R (Yoon et al., 2002), following cycling conditions from Peña et al. (2015). The PCR products were sent to Eurofins Genomics France for bidirectional sequencing using the SANGER method. Sequences were assembled and aligned using CodonCode Aligner® (CodonCode Corporation, United States) and adjusted by eye using SeaView version 5.05 (Gouy et al., 2010); only traces with high quality values and no ambiguities were retained for further analyses. The sequences were 607 bp long.

### Sequences alignment and phylogenetic analyses

**FIGURE 1:**
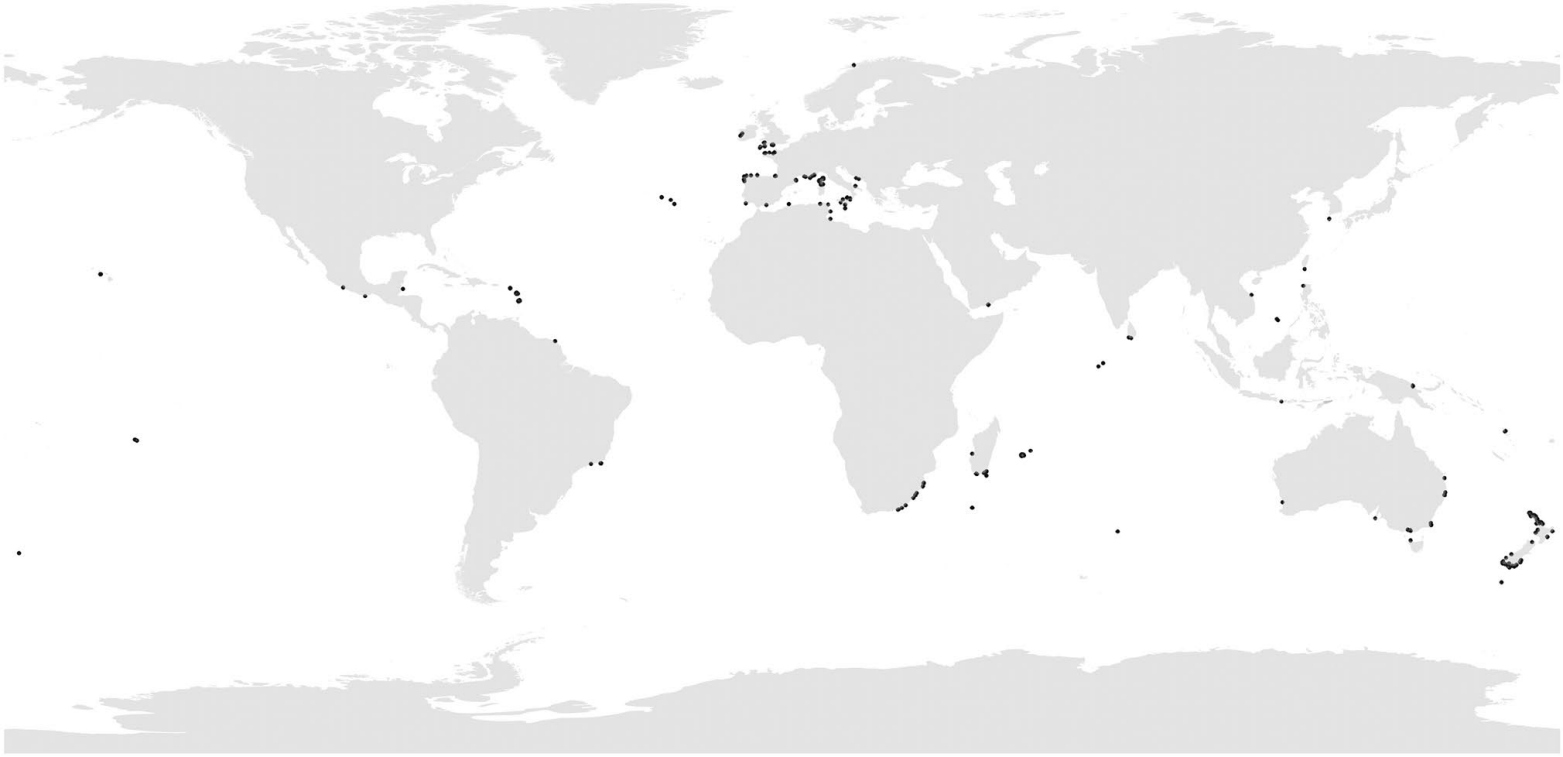
Map of the distribution of the 412 *Jania* specimens studied.

In this study, 138 sequences for the COI and 137 for the *psb*A gene were generated. In addition, a search was done to retrieve *Jania* sequences from GenBank and Barcode of Life Data System (BOLD). We selected, either based on name research or blasting *Jania* sequences, 100 COI and 141 *psb*A gene sequences previously published and assigned to *Jania*, *Cheilosporum*, *Haliptilon*, and *Corallina*. Sequences were aligned using MUSCLE v3.8.31 (Edgar, 2004) as implemented in SeaView. Although model‐based methods can handle missing data, we observed that including very short sequences led to unstable or unreliable placements in gene trees and species‐delimitation analyses. In total, we used sequences of 409 specimens of *Jania* with worldwide distribution (Figure 1). The data set contained 238 COI gene sequences and 278 *psb*A gene sequences. A final data set was built after retaining a single representative sequence per haplotype, with Collapsetypes.pl v4.5 (Chesters, 2013). Phylogenetic analyses were conducted for both COI and *psb*A gene markers, as well as for the two markers concatenated, using maximum likelihood (ML). Before building the concatenated alignment, we checked that the topology inferred from each marker did not show incongruences. The choice of outgroups was made based in light of the phylogeny of corallines from Peña, Bélanger, et al. (2021) and Peña, Harvey, et al. (2021), and our strategy consisted of including the sister taxa to the genus *Jania* for which sequences were available both for the COI and *psb*A genes. We used *Ellisolandia elongata* (JQ615843 for COI, JQ422231 for *psb*A), *Corallina berteroi* (GQ917248 for COI, GQ917438 for *psb*A), *Chiharaea bodegensis* (HM918942 for COI, JQ677011 for *psb*A), and *Calliarthron cheilosporioides* (JQ615594 for COI, JQ422199 for *psb*A). The best model was selected by ModelFinder (Kalyaanamoorthy et al., 2017) for each gene: K81 + F + I + G4 for COI and TIM2 + F + G4 for *psb*A. The concatenated alignment phylogenetic analyses were performed using model partitioning per each region. The ML trees were produced using RAxML‐NG v. 1.1.0 (Kozlov et al., 2019), with selected substitution models, searching 20 trees (10 random and 10 parsimony‐based starting trees) to find the best topology. The MRE‐based bootstopping test was applied to automatically determine the number of bootstrap replicates with the cut‐off value being set to 0.03.

Our COI gene final alignment included 238 sequences among which 119 displayed a unique haplotype. For analysis, the alignment was trimmed to obtain a square matrix of 534 base pairs (bp), with 289 (54%) variable sites, 245 (46%) conserved sites, and 211 (40%) parsimoniously informative characters. From 278 *psb*A gene sequences, 106 unique haplotypes were aligned and trimmed to be 707 bp long, with 220 (31%) variable sites, 487 (69%) conserved sites, and 183 (26%) parsimoniously informative characters. The concatenated alignment used for phylogenetic analyses contained 104 sequences; 63 unique haplotypes were aligned and trimmed to be 1241 bp long, with 395 (32%) variable sites, 846 (68%) conserved sites, and 342 (28%) parsimoniously informative characters. The ML trees were inferred with RAxML; the automatically determined sufficient number of bootstrap replicates was tested with MRE‐based bootstopping, which resulted in 600 bootstrap replicates for the COI gene ML tree and the concatenated gene tree (COI, *psb*A), and 1000 bootstrap replicates for the *psb*A gene ML tree.

### Molecular species delimitation

Two methods were used for species delimitation for both gene data sets (COI and *psb*A): Poisson Tree Process (PTP; Zhang et al., 2013) and Assemble Species by Automatic Partitioning (ASAP; Puillandre et al., 2021). The single PTP (sPTP) modeling was performed with command line mptp v.0.2.5. The ML tree produced by RAxML‐NG was used as input for all PTP analyses. Minimum branch length was detected from FASTA p‐distances for each alignment. Markov chain Monte Carlo (MCMC) support for delimitation was fixed at 100,000. In addition, multirate PTP (mPTP; Kapli et al., 2017) was performed to further assess the confidence of the previous PTP analyses and accounted for the potential divergence in intraspecific diversity and by sampling bias, as mPTP uses one single lambda per coalescent and sPTP uses only one for all. The distance‐based methods of ASAP were conducted on the web server (https://bioinfo.mnhn.fr/abi/public/asap/) based on the p‐distance model. The two best molecular operational taxonomic unit (MOTU) partitions predicted by ASAP were chosen to compare with other methods.

### Biogeography and sea surface temperature

Biogeographical analyses were conducted in R using the shapefile of the Marine Ecoregions of the World (MEOW) as defined by (Spalding et al., 2007) and the coordinates (latitude and longitude) of each specimen to determine to which marine realm it belonged. We also inferred the (sea surface temperature, SST, for each specimen using MARSPEC (Sbrocco & Barber, 2013)).

### Morphological analyses

Images of PC specimens were acquired through BK+ imaging System (Dun, Inc., 2019), equipped with a Canon EOS 5DSR (50 million pixels) with lenses of 100 mm, using magnification of 65 mm and flash. Stacking of images was done by Zerene Stacker, and a scale bar was added with Photoshop pro. Measurements of characters of each sample were made digitally using ImageJ (Schneider et al., 2012). Measurements were collected for five characters—intergenicula length, intergenicula diameter, conceptacle diameter, conceptacle length, and angle of branching—with 10 replicates of each measurement per specimen. Data were collected for 135 specimens in total. These measurements were used together with the realms and SST to attempt species discrimination.

The morphometric data were analyzed with multivariate statistics in RStudio version 2023.12.1 + 402. The morphospace was visualized using a principal components analysis (PCA) with the FactoMineR package (Lê et al., 2008). Discriminant analysis (DA) was carried out to identify the morphological differences between the species using the candisc package (Friendly & Fox, 2021). The morphology of the intergenicula was further analyzed by identifying six landmarks positioned on specific points common to all scales under consideration. The *x*, *y* coordinates of landmark points were digitized and measured by the tps‐Dig program version 2.05 (Rohlf, 2006). Two intergenicula were evaluated per specimen to assess interindividual variability rather than intraindividual variation. Measurement points were placed in the middle of the intergenicula, avoiding basal or apical positions, and whenever possible, on segments without ramification. Geometric morphometric analysis was performed in R using the package geomorph (Adams et al., 2024; Baken et al., 2021) on the landmark coordinates data in order to compare the correlation matrix of the shape of the intergenicula between species, which was reflected in a PCA. Canonical correspondence analysis (CCA) was used to elucidate the relationship among species based on intergenicular characters.

## RESULTS

### Molecular species delimitation

**FIGURE 2:**
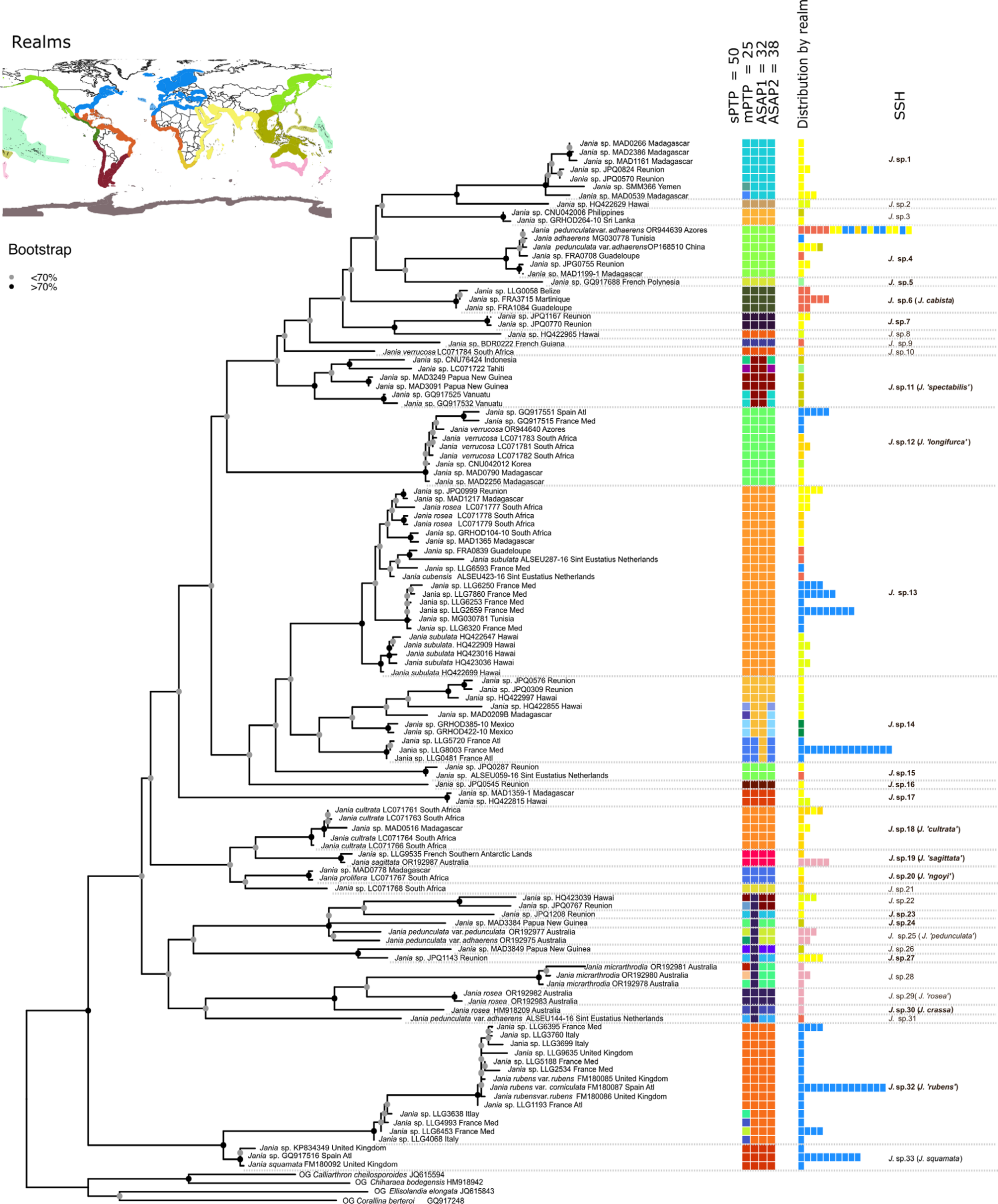
Maximum likelihood tree (RAxML) inferred from the COI gene, showing species delimitation (sPTP, mPTP, ASAP1, and ASAP2). Squares represent specimens sharing the same haplotype, with colors corresponding to their sampling localities based on the marine realms of Spalding et al. (2007; refer to the map on the left). The secondary species hypothesis (SSH) is written on the last column (*n* = 31), in bold, specimens for which morphological analyses were completed in this study.

**FIGURE 3:**
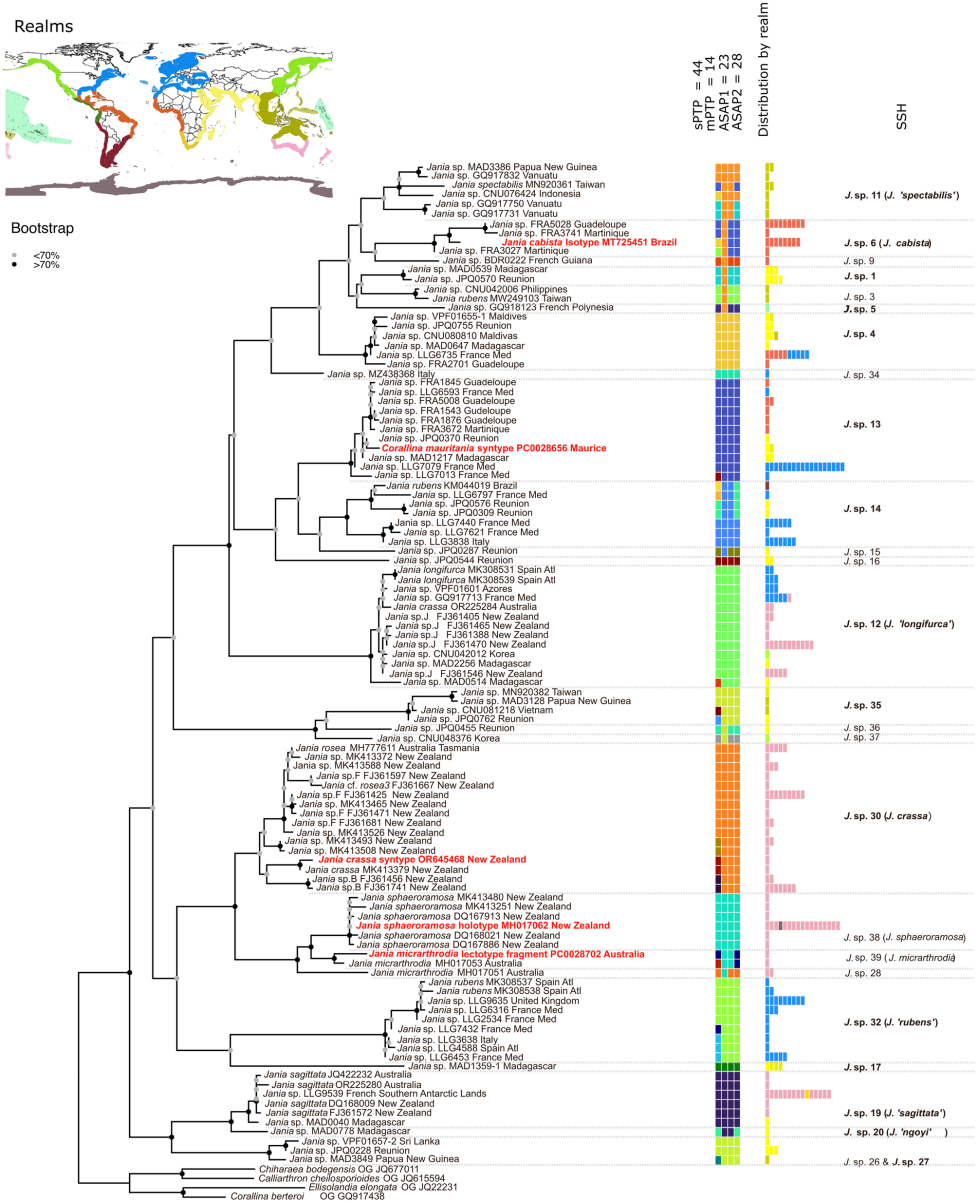
Maximum likelihood tree (RAxML) inferred from the *psb*A gene, showing species delimitation (sPTP, mPTP, ASAP1, and ASAP2). Type specimens are indicated in red. Squares represent specimens sharing the same haplotype, with colors corresponding to their sampling localities based on the marine realms of Spalding et al. (2007; refer to the map on the left). The SSH is written on the last column (*n* = 25), in bold specimens for which morphological analyses were completed.

Phylogenetic trees inferred from the three gene data sets (*psb*A, COI, and *psb*A‐COI combined) are shown respectively in Figure 2, Figure 3 and Figure S1. For each data set, the number of species delimited varied depending on the delimitation methods used. The COI gene data set had the highest number of delimited species regardless of the method used (38–50 putative species; Table 1).

**TABLE 1:** Comparison of the number of delimited species and singletons for each method and data set as well as for each data set, the percentage of species with the same delimitation between two methods, calculated as a proportion of the total number of species in the most conservative method.

Methods/data sets	Number of species delimited|number of singletons			
	sPTP	mPTP	ASAP 1	ASAP 2
COI	50|24	25|7	32|10	38|15
*psb*A	44|18	14|2	23|6	28|7
*psb*A‐COI	24|13	18|16	20|9	19|9

Methods/data sets	Percentage of shared delimited species for COI|*psb*A|*psb*A‐COI			
	sPTP	mPTP	ASAP 1	ASAP 2
mPTP	80%|29%|78%			
ASAP 1	78%|52%|80%	84%|71%|77%		
ASAP2	84%|64%|74%	76%|57%|67%	94%|83%|95%	

Among the delimitation methods used, sPTP identified the highest number of species (up to 50), including the largest number of singleton species. Conversely, mPTP delineated the fewest species; ASAP provided intermediate values (see Table 1). These differences in the number of species delimited using different methods were expected and reflect the sensitivity of the methods, which resulted in greater or lesser splitting within monophyletic groups in the ML tree.

Based on an integrative approach, we proposed secondary species hypotheses (SSHs) by seeking to maximize congruence between molecular delimitations, morphological species, and geographic criteria. In case of discrepancy with any robust criteria allowing a choice between two or more species hypotheses, we opted for the most conservative choice (lumping strategy).

Finally, 39 SSHs were delineated (Figures 2, 3, and Figure S1). Among these 39 SSHs, 20 were represented by both COI and *psb*A gene markers, while 13 (numbered 2, 7, 8, 10, 18, 21 to 25, 29, 31, and 33) and six (34 to 39) were represented only by the COI or *psb*A gene, respectively.

To evaluate the potential contribution of intergenicula morphometric analysis in supporting molecular species delineation, we analyzed a total of 274 measurements, based on six landmarks taken from intergenicula of 137 specimens representing 21 SSHs. Principal component 1 was associated with wedge‐shaped intergenicula, whereas PC2 corresponded to triangular forms. The PCA separated the data into three main groups (Figure S2). The central group comprised 17 species characterized by typically rectangular intergenicula. The two peripheral groups along the *x*‐axis included specimens belonging to species previously classified as *Cheilosporum*; *Jania* sp. 11 (*J. spectabilis*), *J*. sp. 18 (*J. cultrata*), and *J*. sp. 19 (*J. sagittata*), that do not have their type specimens sequenced as well as two other specimens, *J*. sp. 13 and *J*. sp. 27. All species were associated with wedge‐shaped intergenicula ranging from short to elongated forms. It should be noted, however, that these species presented a high degree of intergenicula variability, with measurements also included in the central group.

To complete the morphometric analyses, a DA (Figure S3) was performed using several morphological variables (intergenicular length, diameter, and angle of ramification) as well as one ecological variable (the annual mean SST). The objective was to identify which variables best differentiated the putative species. As expected, species previously classified within *Cheilosporum*, *Jania spectabilis* (*J*. sp. 11), *J. cultrata* (*J*. sp. 18), *J. sagittata* (*J*. sp. 19), *J. ngoyi* (*J*. sp. 20), and *J*. sp. 27 were primarily distinguished from other species based on intergenicular diameter. *Jania cultrata* and *J*. sp. 27 could be differentiated from the other *Cheilosporum*‐like species based on this variable. *Jania* sp. 17, represented by a single specimen, was discriminated from the other species based on its greater length. In contrast, most other species were not clearly distinguished in the DA.

Finally, our results confirmed that intergenicular morphometric and morphological analyses are insufficiently robust for reliable species identification in *Jania*. Only species with wedge‐shaped intergenicula were separated from the other species, but these groupings did not correspond to monophyletic lineages, indicating morphological convergence rather than shared ancestry. Moreover, no SSH could be clearly differentiated based solely on intergenicular variables, with the exception of *Cheilosporum* species.

Assigning names to the molecularly delimited species was particularly challenging due to the difficulty of achieving reliable morphological identification within the genus *Jania*. The most reliable approach was to sequence as many type specimens as possible, including those of synonymized species. When sequencing type material was not possible, specimens collected from the type locality represented a useful alternative, even though morphological comparison with the original type remained necessary.

Of the 39 SSHs delineated (Figures 2, 3, and Figure S1), a species name could only be assigned to 13 SSHs. For four of these, identification was based on sequenced type specimens. For the remaining nine, names were assigned based on previously published morphological identifications and consideration as to whether their geographic distribution encompassed the type locality. In the following, we focus on these 13 identified species, including *Jania* sp. 13, which exhibits a particularly wide distribution spanning four marine realms. For each species, type locality (information from Guiry & Guiry, 2026) and geographic distribution (as realms) from specimens included in this study were indicated.

*Jania cabista*—*J*. sp. 6
Type locality: Brazil
Distribution: Restricted to Tropical Atlantic
Type material: Three isotypes sequenced for the COI and *psb*A genes (Table S1)

**FIGURE 4:**
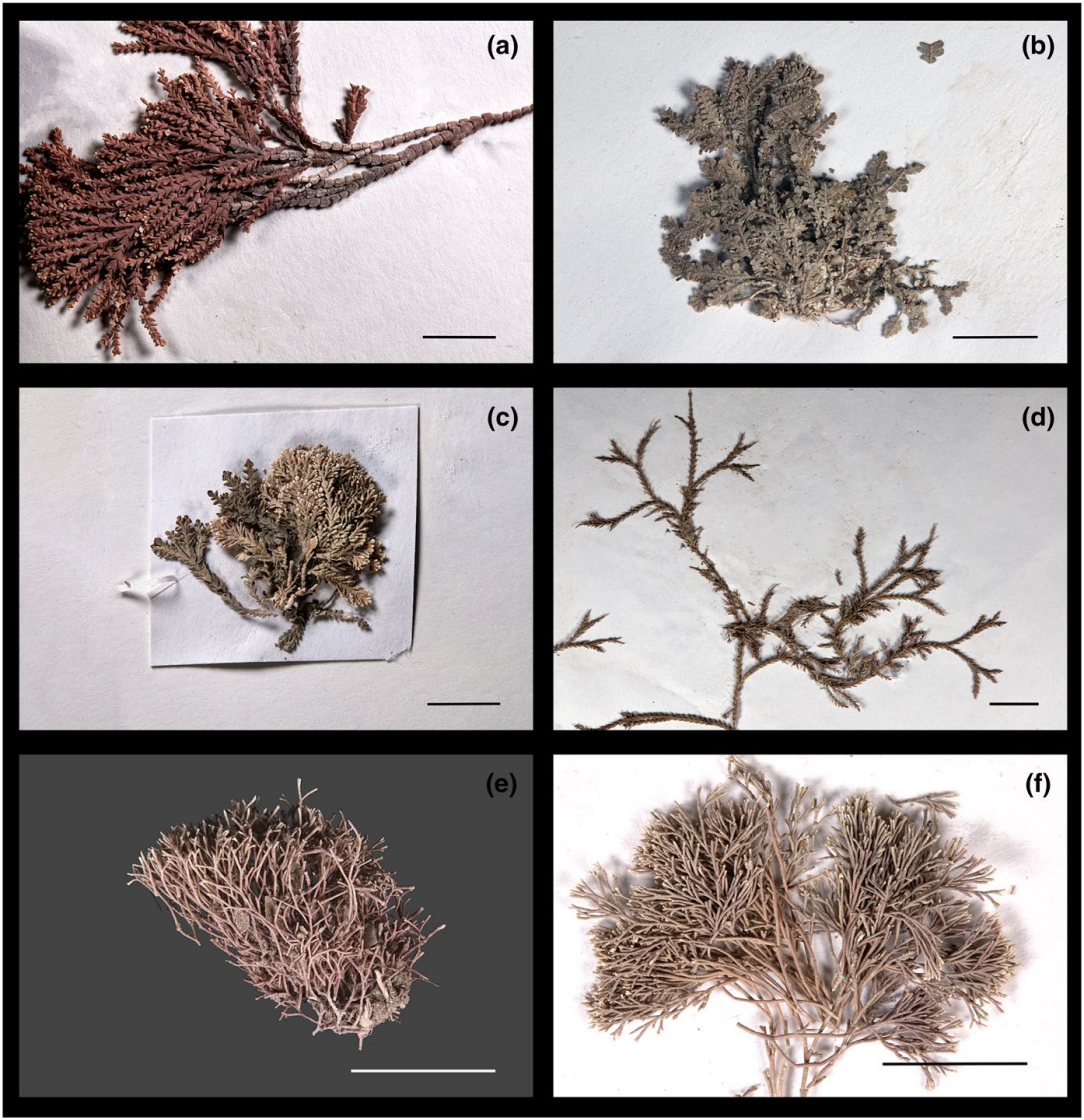
Pictures of specimens delineated as (a) *Jania* *ngoyi* MAD0778; (b) *J*. *spectabilis* MAD3249; (c) *J*. *cultrata* MAD0516; (d) *J*. *sagittata* LLG9539; (e) *J*. *cabista* FRA5028; (f) *J*. *longifurca* MAD2256.

*Jania* sp. 6 was delineated as a single species by most of the analyses (all methods except sPTP in the *psb*A gene and in the combined gene data set). Among the specimens, seven from Brazil were identified as *J. cabista* by Tâmega et al. (2021), including sequences of three isotypes. Consequently, all 12 other specimens of *J*. sp. 6 from the Caribbean (Martinique, Guadeloupe, Belize) were molecularly identified as *J. cabista* (Figures 2 and 3). An example of *J. cabista* is illustrated in Figure 4e.

*Jania crassa*—*J*. sp. 30
Type locality: New Zealand
Distribution: Restricted to Temperate Australasia
Type material: Isolectotype specimen sequenced for the COI gene (Table S1)

*Jania* sp. 30 included specimens with “*rosea*‐type” morphologies (*J*. sp. B, *J*. sp. F.; Farr et al., 2009; Twist et al., 2019) from New Zealand and one specimen from Tasmania (UNB GWS016456) identified as *J. rosea* (Sherwood et al., 2010). However, recently, the *psb*A gene sequence (GenBank OR645468) of the isolectotype of *J. crassa* (PC0028699, type locality New Zealand), published by Nelson et al. (2023), was resolved within the *J*. sp. 30 group, together with GenBank MK413379, also identified as *J. crassa* by Twist et al. (2018). In light of these results, we assigned the name *J. crassa* instead of *J. rosea* to all 39 specimens of *J*. sp. 30 (Figures 2 and 3).

*Jania micrarthrodia—J*. sp. 39, *J. sphaeroramosa*—*J*. sp. 38 *and J*. sp. *28*
Type localities: Australasia (*J. micrarthrodia*), New Zealand (*J. sphaeroramosa*)
Distribution: Restricted to Temperate Australasia
Type material: Lectotype specimen of *J. micrarthrodia* sequenced for the *psb*A gene and type specimen of *J. sphaeroramosa* sequenced for the *psb*A gene (Table S1)

*Jania sphaeroramosa*, *J. micrarthrodia*, and *J*. sp. 28 formed a highly supported monophyletic group comprising 25 specimens in the *psb*A gene data set and only four in the COI gene data set. The phylogenetic tree based on *psb*A gene supported the monophyly of the group, but the four species delimitation methods applied to this data set yielded conflicting results, suggesting between one and four putative species. All sequences except one were previously published by Twist et al. (2018) and Harvey et al. (2020), with specimens identified either as *J. micrarthrodia* (all from Australasian coasts) or as the recently described *J. sphaeroramosa* (all from New Zealand). This group included sequences from two type specimens: the previously published *psb*A gene sequence of *J. sphaeroramosa* (WELT A033619 A + B; GenBank MH017062; type locality: New Zealand; Twist et al., 2018), and a *psb*A gene sequence from the lectotype of *J. micrarthrodia* (PC0028702; type locality: Australasia) obtained in this study (GenBank PX401379). Specimens identified as *J. sphaeroramosa*, including its type, formed a well‐supported monophyletic clade (bootstrap >70%). In contrast, *J. micrarthrodia* was not recovered as monophyletic, as its lectotype specimen sequence was sister to GenBank MH017053, whereas two additional specimens (MH010751 and MH010752, sharing a unique haplotype), previously identified as *J. micrarthrodia*, formed a divergent lineage sister to all remaining specimens of both *J. sphaeroramosa* and *J. micrarthrodia* species. Considering that molecular species delimitation results were not sufficiently conclusive and that both type specimens were clearly distinct and grouped with specimens identified under the same species name, we adopted a conservative approach by recognizing *J. sphaeroramosa* and *J. micrarthrodia* as distinct species. The third, deeply divergent lineage was therefore treated as a putative new species, *J*. sp. 28.

*Jania rosea*—*Jania* sp. 29
Type locality: Southern Seas
Distribution: Restricted to Temperate Australasia

*Jania* sp. 29 included two specimens from Australia identified as *J. rosea* (GenBank OR192982 [LTB 18204], GenBank OR192983 [LTB 18219]; Harvey et al., 2020), sequenced only for the COI gene. This group was close to a specimen UNB GWS016456 (COI gene sequence GenBank HM918209; *psb*A gene sequence GenBank H777611) identified as *J. crassa*. These two groups were delimited as distinct putative species by all methods except mPTP. Additionally, four specimens from South Africa, identified as *J. rosea* (Kogame et al., 2017) were included in another group *J*. sp. 13 with no specimens from Australia. Although the type locality of *J. rosea* is uncertain, the collection from which Lamarck described *Corallina rosea* was registered as “les mers Australes” (southern seas) without additional details about the locality of the type specimen (Harvey et al., 2020). In line with Harvey et al. (2020), who based their identification on the lectotype specimen comparison, we identified *J*. sp. 29 as *J. rosea* (Figures 2 and 3), but the correct application of this name needs to be confirmed by sequencing its lectotype.

*Jania longifurca*—*J*. sp. 12
Type locality: Croatia
Distribution: Temperate Northern Pacific, Temperate Australasia, Temperate Northern Atlantic, Temperate Southern Africa, Western Indo‐Pacific

*Jania* sp. 12 was delineated as one putative species (*n* = 47), except for one specimen from Madagascar PC0166481 (MAD0514), which was delimitated as singleton by sPTP. Within this putative species, five *psb*A gene sequences from specimens from the Atlantic coast of Spain (GenBank MK308531, GenBank MK308533, GenBank MK308534, GenBank MK308535, GenBank MK308539) were morphologically identified as *J. longifurca* (Lugilde et al., 2019; Peña, Bélanger, et al., 2021; Peña, Harvey, et al., 2021). However, five other specimens from South Africa were morphologically identified as *J. verrucosa* by Kogame et al. (2017) and five from Australia as *J. crassa* by Harvey et al. (2020). Since the species name of *J. verrucosa* was proposed to be rejected by Woelkerling, Harvey, and De Reviers (2015a) and the type sequence of *J. crassa* (Nelson et al., 2023) clustered with *J*. sp. 30 (see above), the specimens grouped in this putative species *J*. sp. 12 were assigned to *J. longifurca*. In addition, the presence of four specimens of *J*. sp. 12 collected in the Mediterranean Sea supports the assignment to *J. longifurca*, which has type locality of Croatia but whose type specimen has not been sequenced. An example of *J. longifurca* is illustrated in Figure 4f.

*Jania cultrata*—*J*. sp. 18
Type locality: Port Natal, South Africa
Distribution: Temperate Southern Africa, Western Indo‐Pacific

*Jania* sp. 18 comprised seven specimens from South Africa identified as *J. cultrata* by Kogame et al. (2017), collected near the type locality (Port Natal), as well as two specimens from Madagascar. Based on this evidence, the entire group was identified as *J. cultrata* (Figure 2), whose type specimen has not been sequenced. An example of *J. cultrata* is illustrated in Figure 4c.

*Jania sagittata* – *J*. sp. 19
Type locality: Mauritius
Distribution: Temperate Australasia, Temperate Southern Africa, Western Indo‐Pacific

*Jania* sp. 19 included specimens from New Zealand and Australia identified as *J. sagittata* by Broom et al. (2008), Farr et al. (2009), Hind and Saunders (2013), Twist et al. (2018), Harvey et al. (2020), Peña, Bélanger, et al. (2021); Peña, Harvey, et al. (2021), and Macagnan et al. (2023). Although the type specimen has not been sequenced, one specimen PC0166007 (MAD0040) from Madagascar, which is close to the type locality of *J. sagittata* (Mauritius), was also included in this lineage (Figures 2 and 3). This group was therefore designated as *J*. *sagittata*. An example of *J. sagittata* is illustrated in Figure 4d.

*Jania ngoyi* Maridakis, V.Peña, F.Rousseau & L.Le Gall nom. nov.
Basionym of the replaced name: *Corallina prolifera* J.V.Lamouroux (1816, Histoire des polypiers coralligènes flexibles, vulgairement nommés zoophytes, p. 291, pl. X: fig. 5)
Type locality: East Indies
Distribution: Temperate Southern Africa, Western Indo‐Pacific

Synonyms: *Amphiroa flabellata* Harvey (1849, p. 101). *Amphiroa stangeri* Harvey (1849, p. 101). *Amphiroa heterocladia* Kützing (1858, p. 28). *Cheilosporum pulchellum* Harvey (1855, p. 546). *Cheilosporum proliferum* (J.V.Lamouroux) De Toni (1905). *Cheilosporum africanum* Manza (1937, p. 570).

Etymology: Named in honor of Lilian Masediba Matabane Ngoyi (1911–1980), South African anti‐apartheid activist, in reference to the type locality (South Africa).

Comment: *Jania* sp. 20, with two specimens (two unique haplotypes in the COI gene; one unique haplotype in the *psb*A gene), was delineated as a single species (in the COI gene by all methods; in the *psb*A gene by ASAP2 and sPTP; in the concatenated alignment by all methods except mPTP). One of the specimens (LC071767, temperate Southern Africa) was identified as *Cheilosporum proliferum* by Kogame et al. (2017; Figures 2 and 3). Unfortunately, the comb. nov. *J. prolifera*, proposed by Kim et al. (2007) to transfer *Cheilosporum proliferum* to *Jania*, is illegitimate because the epithet *prolifera* was already used for *Jania prolifera* A.B. Joly 1966: 161, fig. 1; pl. 1: figs 7–9 (Published in: Joly, A.B., Cordeiro, M., Yamaguishi, N. & Ugadim, Y., 1966, New marine algae from southern Brazil. *Rickia* 2: 159–181, VI plates). Following the recommendation 6.11. of the nomenclature code (Madrid Code, Turland et al., 2025), we propose a replacement name *Jania ngoyi* for the illegitimate *Jania prolifera*. An example of *J. ngoyi* is illustrated in Figure 4a. The heterotypic synonyms have been proposed based on morpho‐anatomy but have not been confirmed by DNA sequences.

*Jania squamata*—*J*. sp. 33
Type locality: Western Europe
Distribution: Restricted to Temperate Northern Atlantic

*Jania* sp. 33 comprised specimens previously identified as *J. squamata* by Walker et al. (2009) and Williamson et al. (2015) and collected from Atlantic Spain, United Kingdom, Atlantic France (Figure 2). This distribution is consistent with type locality recorded as “Occidental Europe.”

*Jania rubens* – *J*. sp. 32
Type locality: South of France (*J. rubens* var. *rubens*)
Distribution: Restricted to Temperate Northern Atlantic

*Jania* sp. 32 was largely delimited as a single putative species across both markers (nine unique haplotypes/24 sequences in the *psb*A gene; 14 unique haplotypes/32 sequences in the COI gene; 18 specimens with both gene markers), except by sPTP, which identified multiple species (four in COI and three in *psb*A). In the concatenated tree, unlike ASAP 1 and ASAP 2, which delimited *J*. sp. 32 as one putative species, sPTP and mPTP split it into two putative species. The first group contained specimens distributed in the Mediterranean Sea and along the Atlantic coasts, identified as *J. rubens* (Lugilde et al., 2019; Macagnan et al., 2023; Peña, Bélanger, et al., 2021; Peña, Harvey, et al., 2021) and its varieties *J. rubens* var. *rubens* (Walker et al., 2009) and *J. rubens* var. *corniculata* (Walker et al., 2009). Several specimens from this group showed intergenicula with cornicles on both sides, pointing apically and sometimes developing into two axes. The second group within *J*. sp. 32 was not monophyletic and diverged earlier than the first group, including only specimens from the Mediterranean Sea. Some specimens in this group were corniculate. Among the 22 specimens analyzed for *J*. sp. *32*, seven were distinctly corniculate, while the remaining ones showed varying degrees of cornicles presence, including four specimens without any cornicles. It was also observed that smaller specimens, possibly in early growth stages, exhibited less pronounced cornicles (Figure S4).

Given the molecular species delimitation, the presence of cornicles in specimens of both groups, and without supplementary analysis, we cannot assess the validity of the two varieties *J. rubens* var. *rubens and J. rubens* var. *corniculata*. We therefore considered *J*. sp. 32 as a single putative species identified as *J. rubens* (Figures 2 and 3).

*Jania pedunculata* and *Jania pedunculata* var. *adhaerens* – *J*. sp. *25*
Type localities: Australasia (*J. pedunculata*), the Mediterranean Sea (*J. pedunculata* var. *adhaerens*)
Distribution: Restricted to Temperate Australasia

Specimens identified as *Jania pedunculata* var. *pedunculata* and *J.* *pedunculata* var. *adhaerens* by Harvey et al. (2020) and Macagnan et al. (2023) were resolved here as a single putative species, *J*. sp. 25, by all species delimitation methods except sPTP. The data set included five specimens, all sequenced only for the COI gene. All *J*. sp. 25 specimens were collected in Australia. Other specimens identified as *J. adhaerens* or *J. pedunculata* var. *adhaerens* (Manghisi et al., 2019; Gabriel et al., 2024) were included in another lineage represented by a distinct putative group *J*. sp. 4, which included specimens that had a wide distribution, including the Mediterranean Sea. In addition, one specimen from Sint Eustatius in the Caribbean, identified as *J. adhaerens* (BOLD—ALSEU144‐16), formed a singleton corresponding to *J*. sp. 31. *Jania*. sp. 4 included 36 specimens characterized by high haplotype diversity (six unique haplotypes/19 *psb*A; six unique haplotypes/27 in COI). Species delimitation methods consistently identified this group as a single putative species. Specimens assigned to *J*. sp. 4 had a diverse distribution: Maldives, Guadeloupe, Reunion, Malta, South Africa, Croatia, France, Madagascar, Tunisia, Taiwan, Italy, China, Azores, and Yemen (Figure 2).

The Australian specimens of *Jania*. sp. 25, identified as *J. pedunculata* var. *pedunculata* and *J. pedunculata* var. *adhaerens*, originate from the same region as the type locality of *J*. *pedunculata* var. *pedunculata* and were identified by Harvey et al. (2020) based on morphological comparison with type material. In the data set, no other specimens were identified *as J. pedunculata*. Therefore, we attributed the name *J. pedunculata* to the specimens in *J*. sp. *25*. However, as other specimens identified as *J*. *pedunculata* var. *adhaerens* were delineated in three different species, we could not confirm the identity of this variety, without sequence data from type material.

*Jania spectabilis*—*J*. sp. 11
Type locality: Tonga Pacific
Distribution: Central Indo‐Pacific, Eastern Indo‐Pacific

*Jania* sp. 11, comprised 13 specimens (six unique haplotypes/eight sequences in *psb*A; six unique haplotypes/six sequences in COI; three specimens with both sequences). This group was delimited as a single species by ASAP 1 (COI and *psb*A), mPTP (COI), and all methods in the concatenated tree, except sPTP. One specimen from Tahiti (GenBank LC071722) was identified as *J. spectabilis* by Kogame et al. (2017). Given that the other specimens were also from the Indo‐Pacific (Papua New Guinea, Taiwan, Vanuatu, and Indonesia), and that most of the methods delineated *J*. sp. 11 as a one species, we tentatively assigned the name *J. spectabilis* to all the specimens in this group (Figures 2 and 3). An example of *J*. *spectabilis* is illustrated in Figure 4b.

*Jania* sp. 13
Distribution: Tropical Atlantic, Temperate Northern Atlantic, Western Indo‐Pacific, Indo‐Pacific Warm Water, Temperate Southern Africa

*Jania* sp. 13 was delineated as a single species by all methods for both genes (except for one specimen, PC0626298 (LLG7013), delimited as a different species by sPTP in *psb*A) and for the concatenated alignment. It is represented by 59 specimens with high haplotype diversity (11 unique haplotypes over 32 sequences in *psb*A and 22 unique haplotypes over 44 sequences in COI; 19 specimens with both sequences). The two type specimens *Corallina polydactyla*, lectotype PC0028626 (type locality: Reunion Island) and *C. mauritiana* syntype PC0028656 (type locality Mauritius), displayed identical sequences that grouped within *J*. sp. 13. *Corallina polydactyla* and *C. mauritiana* were considered synonyms of *Haliptilon polydactylum and H. mauritianum*, respectively. Knowing that *Haliptilon* is congeneric with *Jania* according to Kim et al. (2007), both species should be transferred to *Jania*. As the nomenclatural transfer has not been published, the taxonomic or nomenclatural status of these two type specimens is considered unresolved (Guiry & Guiry, 2026).

*Jania* sp. 13 also included one specimen from Saint Eustatius in the Caribbean identified as *J. cubensis* (type locality: Cuba) (*Haliptilon cubensis*; ALSEU423‐16—BOLD—L.M. van der Loos 2015), seven specimens from Hawaii identified as *J. subulata* (basionym: *Haliptilon subulatum*, type locality: West Indies) by Sherwood et al. (2010; Figures 2 and 3).

In the studied specimens (from PC collection), only the putative species *Jania* sp. 13 had pinnate ramification, a characteristic of *Haliptilon*‐like species. The primary ramification was dichotomous, and the secondary ramification was pinnate. The intergenicula within specimens of this group varied significantly in shape and size. Some specimens had flat and wider intergenicula distally (winged‐like); others were more terete. The diameter of the intergenicula varied from 70 μm to 600 μm (30–1100 μm); 77% of the specimens had an intergenicular diameter under 200 μm, and 33% had a diameter between 300 and 600 μm. Intergenicula length varied between 460 μm and 740 μm (250–1000 μm; Figure S5).

Among the previously recognized *Haliptilon* species, six are currently accepted, including two, *Jania squamata* and *J. rosea*, which were represented in our molecular data set as distinct lineages. *Jania* sp. 13 may correspond to one of the four remaining accepted names: *J. subulata*, *J. cubensis*, *J. virgata*, or *J. paniculata*. Of these, *J. subulata* is the oldest validly published name (originally described as *Corallina subulata* in 1786), predating both *H. polydactylum* (1862) and *H. mauritianum* (1943). However, assigning the name *J. subulata* to *Jania* sp. 13 remains premature given the limited number of reliably identified specimens currently available. Several specimens in our data set were identified as *J. subulata* (eight specimens in six unique haplotypes) from Hawaii and from the Caribbean (the type locality is in the West Indies). Given the potential taxonomic implications, with the synonymization of several species, additional sampling from the type localities as well as molecular data from type material of *J. subulata* and other *Haliptilon*‐like species appears necessary before any formal taxonomic reassignment can be made.

## DISCUSSION

### Species delimitation within the genus *Jania*

We investigated species diversity within *Jania* using an integrative approach confronting molecular delimitation tools to morpho‐anatomical and geographic data. Our results confirmed that species identification and delimitation based solely on morpho‐anatomical characters alone remain problematic. The limitation of traditional characters is well known: Some characters, such as reproductive features, are not always present; phenotypic variability and plasticity cannot be reliably correlated with environmental conditions and life‐history traits; character states often overlap; and certain analyses require lengthy decalcification processes (see Harvey et al., 2020 and their 79‐character data set). Therefore, molecular analyses appeared essential for robust species delimitation, providing a reliable framework to reassess *Jania* diversity and to evaluate the relevance of specific morphological characters. In this study, we focused on easily accessible vegetative characters, specifically intergenicula measurements, obtained through automated analysis of an image bank. Statistical analyses of these measurements compared with molecular delimitations confirmed that these traits are not sufficiently discriminant to be used alone for species identification and delimitation in *Jania*. They did, however, allow for the differentiation of species previously assigned to *Cheilosporum* (notably species 11, 18, 19, 27, and possibly 20) from other species of *Jania*. In contrast, species formerly classified under *Haliptilon* (characterized by pinnate branching) present a more complex situation, as their measurements do not clearly distinguish them. Nonetheless, it is noteworthy that *J*. sp.13, *Haliptilon*‐like, exhibits high morphological variability.

In our study, 39 SSHs were delimited, whereas 54 species are currently accepted (Guiry & Guiry, 2026) in *Jania*. Therefore, the number of putative species delimited in our study is lower than the number of accepted *Jania* species, a result that somewhat contrasts to what has been encountered for other coralline algae for which recent studies have detected cryptic and pseudo‐cryptic species based on DNA sequences combined with morpho‐anatomical observations (Brodie et al., 2021; Lugilde et al., 2019; Pardo et al., 2015; Tâmega et al., 2021; Twist et al., 2018).

Molecular delimitation without reliable morpho‐anatomical identification still faces the challenge of assigning a species name to molecular species hypotheses. To overcome this issue, the best practice is to sequence type specimens in order to bridge the gap between type‐bearing names and DNA sequences. Unfortunately, this optimal approach is not always feasible, as type specimens are not always available, and the approach requires the destruction of a small fraction of the type material for DNA extraction, which is not always permitted by collection curators. An alternative is to sequence specimens from the type locality and identify them using morpho‐anatomical characters in light of the actual type specimens. However, this topotype approach has its own limitations: Localities of type specimens can be so vague that they are not helpful, or the species may no longer be present in its original type locality due to distribution shifts caused by global changes (e.g., climate warming, land‐use changes, etc.). Last but not least, multiple *Jania* species may coexist sympatrically, leading to potential misidentifications.

### Species delimitation and identification

#### Jania rubens

*Jania* *rubens* is the generitype of *Jania*. Linnaeus (1758) described *Corallina rubens* as dichotomous, thread‐like, with cylindrical, extremely short joints (intergenicula), and subclavate dichotomies. Unfortunately, no complementary illustrations or detailed morpho‐anatomical studies of the lectotype have been published previously at this time. Since then, *J. rubens* has been recorded from numerous localities in the Mediterranean Sea, the Atlantic Ocean, and the Indo‐Pacific Ocean; however, many of these records require confirmation.

For example, based on morphological characteristics, specimens of *Jania rubens* from southern Australia were misidentified specimens of *J. micrarthrodia* (Harvey et al., 2020). Based on our molecular results, specimen SPF 57696 (GenBank KM044019; Torrano‐Silva et al., 2014) from Brazil, identified as *J*. rubens, appears to be delineated as *J*. sp. 14. In our results, *J. rubens* was delineated as a single putative species with a distribution restricted to the Temperate Northern Atlantic and Mediterranean coasts, with the type locality being in the Mediterranean Sea, south of France. The putative species *J. rubens* was either recovered as a single species or split into two species, the latter scenario being supported only by PTP methods applied to the concatenated alignment differentiating *J. rubens* into two species. One group was exclusively distributed in the Mediterranean, while the other was observed in both the Mediterranean and Atlantic coasts. Both groups were consistent with the type locality in south of France. Morphologically, these two groups could not be distinguished based on intergenicular measurements, which ranged from 45 to 170 μm in width and 300 to 1000 μm in length. The observed conflict among molecular delimitation methods and data sets suggests that we may be reaching the resolution limits of current analytical approaches. The use of more variable genetic markers could help clarify whether *J. rubens* should be split into two distinct species. *Jania rubens* includes three to seven currently accepted varieties (Guiry & Guiry, 2026). To assess whether one of the two lineages might correspond to *J. rubens* var. *corniculata*, we examined all available specimens from both groups for the presence of cornicles. Linnaeus (1758) described *Corallina cornicula* based on the presence of “bicornibus” intergenicula as a diagnostic character. Cornicles were observed in specimens distributed across both groups. Given that neither molecular delimitation nor morpho‐anatomical or geographic data provided clear support for species‐level separation, we chose to treat *Jania rubens* as a single species. Nonetheless, the presence of both corniculate and non‐corniculate specimens in each lineage renders this character non‐diagnostic, which constitutes an argument toward the synonymization of *J*. *rubens* var. *corniculata* with *J. rubens*.

#### *Jania sphaeroramosa*, *J. micrarthrodia*, and *J.* sp. 28

In our results, the *psb*A gene analyses revealed that *Jania sphaeroramosa* and *J. micrarthrodia* were distinct groups, characterized by a distribution corresponding to their type locality (respectively New Zealand and Australia) and the presence of both type specimen: the holotype of *J. sphaeroramosa* and the lectotype of *J. micrarthrodia*. However, both groups were molecularly delineated as one species by two of the four methods used (ASAP1 and mPTP), which makes the separation of *J. sphaeroramosa* from *J. micrarthrodia* based on molecular results questionable. Additional specimens and genetic markers are needed to clarify whether the three putative species, including *J. micrarthrodia* and *J. sphaeroramosa*, should be synonymized, and whether *J*. sp. 28 represents a distinct species or falls within *J. micrarthrodia*.

#### *Jania crassa* and *J. rosea*

Farr et al. (2009) distinguished two morphologically distinct forms among specimens identified as *Jania rosea* from New Zealand, the “feather” and “bottlebrush” morphotypes, which they designated as *J*. sp. F and *J*. sp. B, respectively. Molecular analyses by Macagnan et al. (2023) confirmed the high morphological and genetic variability of *J*. *rosea* and supported its recognition as a species complex. In our results, specimens morphologically identified as *J. rosea* by Farr et al. (2009), including both the feather and bottlebrush morphotypes from New Zealand and Tasmania, were delineated as *J*. sp. 30 (*J. crassa*), which also included the isolectotype of *J. crassa* (*psb*A gene: OR645468–PC0028699). In contrast, Australian specimens identified as *J. rosea* by Harvey et al. (2020) were assigned to *J*. sp. 29 (*J*. *rosea*), which was only included in the COI gene phylogeny. These two lineages (*J. crassa* and *J*. *rosea*) are not monophyletic, since both representatives of *J. rosea* were sister group to *J*. sp. 39 (*J. micrarthrodia*). Sequencing representatives of all the four putative species (*J. crassa*, *J*. *rosea*, *J*. *micrarthrodia*, and *J*. *sphaeroramosa*) for both COI and *psb*A gene markers appears necessary to clarify the relationships and better delineate species within this large group restricted principally to temperate Australasian coasts. Three type specimens have been already sequenced for the *psb*A gene, but the sequence of the type of *J*. *rosea* is still mandatory to unequivocally assign the names *J. crassa* and *J*. *rosea*.

#### *Jania pedunculata* var*. adhaerens*

In 2020, Harvey et al. proposed the inclusion of *Jania adhaerens* as a variety of *J. pedunculata*, based exclusively on vegetative morphological characters. Molecular results (Macagnan et al., 2023) of the specimens identified by Harvey as *J. pedunculata* var. *pedunculata* and *J. pedunculata* var. *adhaerens* were all grouped under *J*. sp. 25 (*J. pedunculata*) restricted to Australia. Conversely, other specimens previously identified as *J. pedunculata* var. *adhaerens* (or *J. adhaerens*) were recovered in two distinct SSHs: one represented by a singleton from the Caribbean (*J*. sp. 31) and the other (*J*. sp. 4) comprising specimens from widely separated localities, including Tunisia (Manghisi et al., 2019), China (GenBank: Bao et al. 2022), and the Azores (Gabriel et al., 2024). *Jania* sp. 4 is geographically widespread occurring across multiple biogeographic realms, such as the Western Indo‐Pacific, Central Indo‐Pacific, Tropical Atlantic, Temperate Northern Atlantic, Temperate Southern Africa, and Indo‐Pacific Warm Water. Interestingly, it was not detected in Temperate Australasia. These results raise the question of whether *J*. sp. 4 may in fact correspond to *J. adhaerens* sensu stricto. The type locality of *J. pedunculata* var. *adhaerens* is uncertain; Lamouroux's original French label mentions “Méditerranée?”, although this locality was omitted in the English translation (Harvey et al., 2020). Notably, our sampling of *J*. sp. 4 included specimens from several Mediterranean locations (France, Italy, Tunisia, Malta, and Croatia), consistent with a possible Mediterranean type locality. Morphological comparisons between our *J*. sp. 4 specimens and the lectotype of *J. adhaerens* (as described by Harvey et al., 2020) revealed a high degree of similarity. Key traits, including branching angles (lectotype of *J. adhaerens*: 20–90°; *J*. sp. 4: 18–115°, mean 55°, *SD* 6°), intergenicular lengths (lectotype of *J. adhaerens*: 250–800 μm; *J*. sp. 4: 190–1100 μm), and widths (lectotype of *J. adhaerens*: 70–140 μm; *J*. sp. 4: 35–183 μm), overlapped considerably. Both also shared the presence of multiple small holdfasts and apical non‐fertile intergenicula that were rounded or pointed. The main morphological character used by Harvey et al. (2020) to distinguish the two varieties of *J. pedunculata* was the shape of the apical vegetative intergenicula: swollen (cuneiform, pyriform, or globular) in *J. pedunculata* var. *pedunculata*, and consistently columnar in *J. adhaerens*. However, columnar apical intergenicula are commonly observed across many *Jania* species, making them of limited diagnostic value. In contrast, swollen intergenicula are relatively uncommon and might appear more distinctive. This discrepancy highlights the limitations of using vegetative morphology alone, especially those features potentially influenced by environment or developmental factors, in delineating species boundaries. Our morphometric analyses further confirmed that *J*. sp. 4 cannot be distinguished from other species using intergenicular dimensions alone, reinforcing the need for integrative approaches combining morphology with molecular data. Clarifying the identity of *J*. *pedunculata* var. *adhaerens* will require sequencing the type material. Such analysis could determine whether it is conspecific with *J. pedunculata*, as proposed by Harvey et al. (2020) or, instead, corresponds to *J*. sp. 4, a distinct and widely distributed species absent from Australasia.

### Species diversity of *Jania* in Europe and in the Caribbean

The specimens sequenced in this study were widely distributed, but metropolitan and overseas French territories were particularly well represented thanks to the Muséum program “Our reviewed Planet,” which conducts inventories of biodiversity.

#### Focus on temperate European species

According to AlgaeBase, 17 species of *Jania* have a European distribution (Guiry & Guiry, 2026). A revision of the species of *Jania* in Atlantic Iberia, the French Atlantic, and the British Isles listed four taxa (Lugilde et al., 2016, 2017): *J. longifurca*, *J. rubens*, *J. rubens* var. *corniculata*, and *J. squamata*. In their checklist of *Jania* from Galicia, Lugilde et al. (2019) reported a fifth species, *J. virgata*, which was less common in the region with only older records. In the Mediterranean Sea, Cormaci et al. (2020) listed six species: *J. rubens* var. *rubens*, *J. squamata*, *J. longifurca*, *J. pedunculata* var. *adhaerens*, *J. rubens* var. *corniculata*, and *J. virgata*.

We delineated seven putative species with a European distribution (temperate northern Atlantic), three identified as *Jania* *rubens* (Mediterranean and Atlantic), *J*. *squamata* (Atlantic), *J*. *longifurca* (Macaronesia, Mediterranean, Atlantic), and four species not yet identified: *J*. sp. 4 (Macaronesia, Mediterranean), *J*. sp. 13 (Mediterranean), *J*. sp. 14 (Macaronesia, Mediterranean, Atlantic), and the singleton GenBank MZ438368 from Italy (Mediterranean).

#### Focus on Caribbean species

According to AlgaeBase (Guiry & Guiry, 2026), 10 species and subspecific taxa of *Jania* are reported in the Caribbean Sea. These include *J. cubensis*, *J. subulata*., *J. pedunculata* var. *adhaerens*, *J. caespitosa*, *J. capillacea* Harvey, *J. continua*, *J. lesueri*, *J. pumila*, *J. rubens*, and *J. cabista*. Various inventories of macroalgae have been conducted in the West Indies. For example, in the Bahamas, Ballantine and Aponte (2005) recorded *J. pedunculata* var. *adhaerens* and *J. cubensis*. In Barbados, Wynne et al. (2014) identified six species: *J. pedunculata* var. *adhaerens*, *J. capillacea*, *J. cubensis*, *J. pumila*, *J. rubens*, and *J. subulata*. These same species were also listed as in Cuba (Suarez et al., 2017). In the fifth revision of marine algae of the tropical and subtropical Western Atlantic, Wynne (2022) listed 19 species of *Jania*, including *J. cabista*, *J. caespitosa*, *J. capillacea*, *J. continua*, *J. cubensis*, *J. pedunculata var. adhaerens*, *J. prolifera*, *J. pumila*, *J. rosea*, *J. rubens, J. subulata*, *J. lesueri*, *J. crassa, J. cultrata*, *J. huertae* (unresolved), *J. sanctae‐marthae*, *J. sagittata*, *J. tenella*, and *J. ungulata* f. *brevior*. Herein, we delineated six putative species in the Caribbean islands: *J*. sp. 15, *J*. sp. 31 (singleton), *J*. sp. 13, *J*. sp. 4, and *J*. sp. 6 (*J. cabista*).

Our results once again identified fewer species than the currently accepted or listed as from both Europe and the Caribbean. However, in the Caribbean, our sampling was not exhaustive and was primarily concentrated in the Eastern Caribbean, specifically in the French West Indies (Martinique and Guadeloupe). The six species we identified are close in number to the five species listed in the macroalgal checklist of Martinique by Delnatte and Wynne (2016): *Jania pedunculata* var. *adhaerens, J. capillacea, J. cubensis, J. rubens*, and *J. subulata*.

### Distribution of the genus *Jania*

Based on our results, the species of *Jania* have mostly a distribution restricted to a single realm, 18 putative species were tropical and 12 species temperate. However, according to our results, three putative species were from the same ocean, whereas five putative species had a wider distribution. Among these latter species, three (*J*. *longifurca*, *J*. sp. 4, and *J*. sp. 13) had haplotype diversity, suggesting that they have higher capacities for dispersion than the other species of *Jania*. In the case of *J. longifurca*, it has a great ability to attach to various substrates and materials in maerl beds through secondary discs and calcified thickenings and has often been observed without reproductive structures, suggesting a strategy of propagation by fragmentation (Lugilde et al., 2019). Unfortunately, our knowledge of the biology of *Jania* remains limited, and we are still far from fully understanding its life‐history traits. Our results suggest that contrasting reproductive strategies—such as vegetative multiplication versus sexual reproduction—as well as differences in spore dispersal capacity are at play in this genus.

## CONCLUSIONS

Using a representative collection of *Jania* specimens, we delineated only 39 species, whereas 54 have been formally accepted. We confirmed the extensive distribution of *Jania*, with some lineages being restricted to specific regions (Temperate North Atlantic, Temperate Australasia) whereas others have wider distributions. Notably, three species were globally distributed and had high haplotype diversity. *Jania* *longifurca*, *J*. sp. 4, and *J*. sp. 13 had different species identifications, including two based on sequences of type specimens (*Corallina polydactyla* Montagne & Millardet 1862, lectotype PC0028626, *and C. mauritiana* Børgesen 1943, syntype PC0028656). *Jania* sp. 13 displayed high morphological diversity, also observed in *J. longifurca* (Lugilde et al., 2019), with the distinctive ‘*Haliptilon*‐like’ ramification pattern. Species previously assigned to the genera *Cheilosporum* and *Haliptilon* were not resolved as monophyletic. Additional sequences from type specimens would provide an opportunity to bridge the gap between type‐bearing names and molecular sequences. Even if our study presents a comprehensive sampling of *Jania*, it is only a first step, and additional samples from key geographic zones, including regions not covered so far, as well from as type localities, would resolve ties among species in order to assess the genuine diversification and dispersion of the species of *Jania*.

## AUTHOR CONTRIBUTIONS

**Clio Maridakis:** Conceptualization (equal); data curation (lead); formal analysis (lead); investigation (lead); visualization (equal); writing – original draft (lead); writing – review and editing (equal). **Viviana Peña Freire:** Data curation (equal); writing – review and editing (equal). **Gilberto Marani:** Data curation (equal). **Maria Tarin Sancho:** Data curation (equal); formal analysis (equal). **Line Le Gall:** Conceptualization (equal); data curation (equal); funding acquisition (equal); investigation (equal); resources (equal); supervision (equal); writing – review and editing (equal). **Florence Rousseau:** Conceptualization (equal); data curation (equal); funding acquisition (equal); investigation (equal); resources (equal); supervision (equal); writing – review and editing (equal).

## Supporting information


**Figure S1.** Maximum likelihood tree (RAxML) inferred from concatenated gene alignment *psb*A‐COI; showing species delimitation (sPTP, mPTP, ASAP1, and ASAP2). Squares represent specimens sharing the same haplotype, with colors corresponding to their sampling localities based on the marine realms of Spalding et al. (2007; refer to the map on the left). The SSH is written on the last column (*n =* 20); in bold specimens for which morphological analyses were completed.
**Figure S2.** Principal component analysis of the shape of the intergenicula (*n* = 2 per specimen) for 136 specimens of 21 SSHs. The first two principal components accounted for 89.38% of the total variation (PC1 = 78.81%, PC2 = 10.58%). Number of specimens per species: *Jania cabista* (*n* = 10), *J. crassa* (*n* = 1), *J. cultrata* (*n* = 2), *J. longifurca* (*n* = 7), *J. ngoyi* (*N* = 1), *J. rubens* (*N* = 21), *J. sagittata* (*n* = 2), *J*. sp. 1 (*n* = 8), *J*. sp. 4 (*n* = 13), *J*. sp. 5 (*N* = 1), *J*. sp. 7 (*N* = 1), *J*. sp. 13 (*N* = 32), *J*. sp. 14 (*N* = 20), *J*. sp. 15 (*N* = 1), *J*. sp. 16 (*N* = 1), *J*. sp. 17 (*N* = 4), *J*. sp. 23 (*N* = 1), *J*. sp. 24 (*N* = 1), *J*. sp. 27 (*N* = 4), *J*. sp. 35 (*N* = 1), and *J. spectabilis* (*n* = 5).
**Figure S3.** Discriminant analysis (DA) of morphological characters and environmental data for 136 specimens representing 21 SSHs. Per specimen, the following mean values were used: intergenicular length (IL, *n* = 10), intergenicular diameter (ID, *n* = 10), angle of ramification (IA, *n* = 10), and mean annual temperature (*T*mean). The two discriminant axes explained 85% of the total variation. For putative species with multiple specimens, circles indicate the spread of samples. Sample sizes per species: *J. cabista* (*n =* 10), *J. cultrata* (*n =* 2), *J. longifurca* (*n =* 6), *J. ngoyi* (*n =* 1), *J. rubens* (*n =* 19), *J. sagittata* (*n =* 3), *J*. sp. *1* (*n =* 8), *J*. sp. *13* (*n =* 31), *J*. sp. *14* (*n =* 20), *J*. sp. *15* (*n =* 1), *J*. sp. *16* (*n =* 1), *J*. sp. *17* (*n =* 4), *J*. sp. *23* (*n =* 1), *J*. sp. *24*(*n =* 1), *J*. sp. *27* (*n =* 4), *J*. sp. *4* (*n =* 12), *J*. sp. *7* (*n =* 1), and *J. spectabilis* (*n =* 4). The biplot shows separation of some SSH, primarily along the first discriminant axis (*x*‐axis). However, many SSHs cluster closely, indicating limited discrimination. Intergenicular diameter is strongly correlated with the *x*‐axis, whereas intergenicular length correlates with a diagonal axis running from the top left to the bottom right of the plot.
**Figure S4.** Pictures of specimens delineated as *Jania* *rubens*; the arrow in indicated cornicles. Pictures A and B are specimens only from the Mediterranean and pictures C to E are representing specimens from the Mediterranean and the Atlantic A_LLG2729; B_LLG6453; C_LLG3639; D_LLG5198; E_LLG6395.
**Figure S5.** Pictures of specimens delineated as *Jania* sp. 13; the arrow is indicating the pinnate ramification. A_FRA0839; B_ LLG2659; C_LLG6253; and D_MAD1365.

**Table S1.** List of the sequences from GB and BOLD, and from the specimens sequenced from the collection PC.
