# Supplementary material for: Are integrative systematic tools efficient toward unraveling species diversity with the genus Jania (Corallinaceae, Rhodophyta)?
Source: J Phycol. 2026 Apr 29;62(3):781–800. doi: 10.1111/jpy.70167 (PMC13280778; doi:10.1111/jpy.70167)
Supplement: Supplementary file 1 — Figure S1. Maximum likelihood tree (RAxML) inferred from concatenated gene alignment psbA‐COI; showing species delimitation (sPTP, mPTP, ASAP1, and ASAP2). Squares represent specimens sharing the same haplotype, with colors corresponding to their sampling localities based on the marine realms of Spalding et al. (2007; refer to the map on the left). The SSH is written on the last column (n = 20); in bold specimens for which morphological analyses were completed. Figure S2. Principal component analysis of the shape of the intergenicula (n = 2 per specimen) for 136 specimens of 21 SSHs. The first two principal components accounted for 89.38% of the total variation (PC1 = 78.81%, PC2 = 10.58%). Number of specimens per species: Jania cabista (n = 10), J. crassa (n = 1), J. cultrata (n = 2), J. longifurca (n = 7), J. ngoyi (N = 1), J. rubens (N = 21), J. sagittata (n = 2), J. sp. 1 (n = 8), J. sp. 4 (n = 13), J. sp. 5 (N = 1), J. sp. 7 (N = 1), J. sp. 13 (N = 32), J. sp. 14 (N = 20), J. sp. 15 (N = 1), J. sp. 16 (N = 1), J. sp. 17 (N = 4), J. sp. 23 (N = 1), J. sp. 24 (N = 1), J. sp. 27 (N = 4), J. sp. 35 (N = 1), and J. spectabilis (n = 5). Figure S3. Discriminant analysis (DA) of morphological characters and environmental data for 136 specimens representing 21 SSHs. Per specimen, the following mean values were used: intergenicular length (IL, n = 10), intergenicular diameter (ID, n = 10), angle of ramification (IA, n = 10), and mean annual temperature (Tmean). The two discriminant axes explained 85% of the total variation. For putative species with multiple specimens, circles indicate the spread of samples. Sample sizes per species: J. cabista (n = 10), J. cultrata (n = 2), J. longifurca (n = 6), J. ngoyi (n = 1), J. rubens (n = 19), J. sagittata (n = 3), J. sp. 1 (n = 8), J. sp. 13 (n = 31), J. sp. 14 (n = 20), J. sp. 15 (n = 1), J. sp. 16 (n = 1), J. sp. 17 (n = 4), J. sp. 23 (n = 1), J. sp. 24(n = 1), J. sp. 27 (n = 4), J. sp. 4 (n = 12), J. sp. 7 (n = 1), and J. spe [file JPY-62-781-s002.docx]

**FIGURE S1.** ML tree (RAxML) inferred from concatenated alignment psbA-COI; showing species delimitation (sPTP; mPTP; ASAP1; ASAP2). Squares represent specimens sharing the same haplotype, with colors corresponding to their sampling localities based on the marine realms of Spalding et al. (2007) (refer to the map on the left). The SSH is written on the last column (n=20); in bold specimens for which morphological analyses were completed.


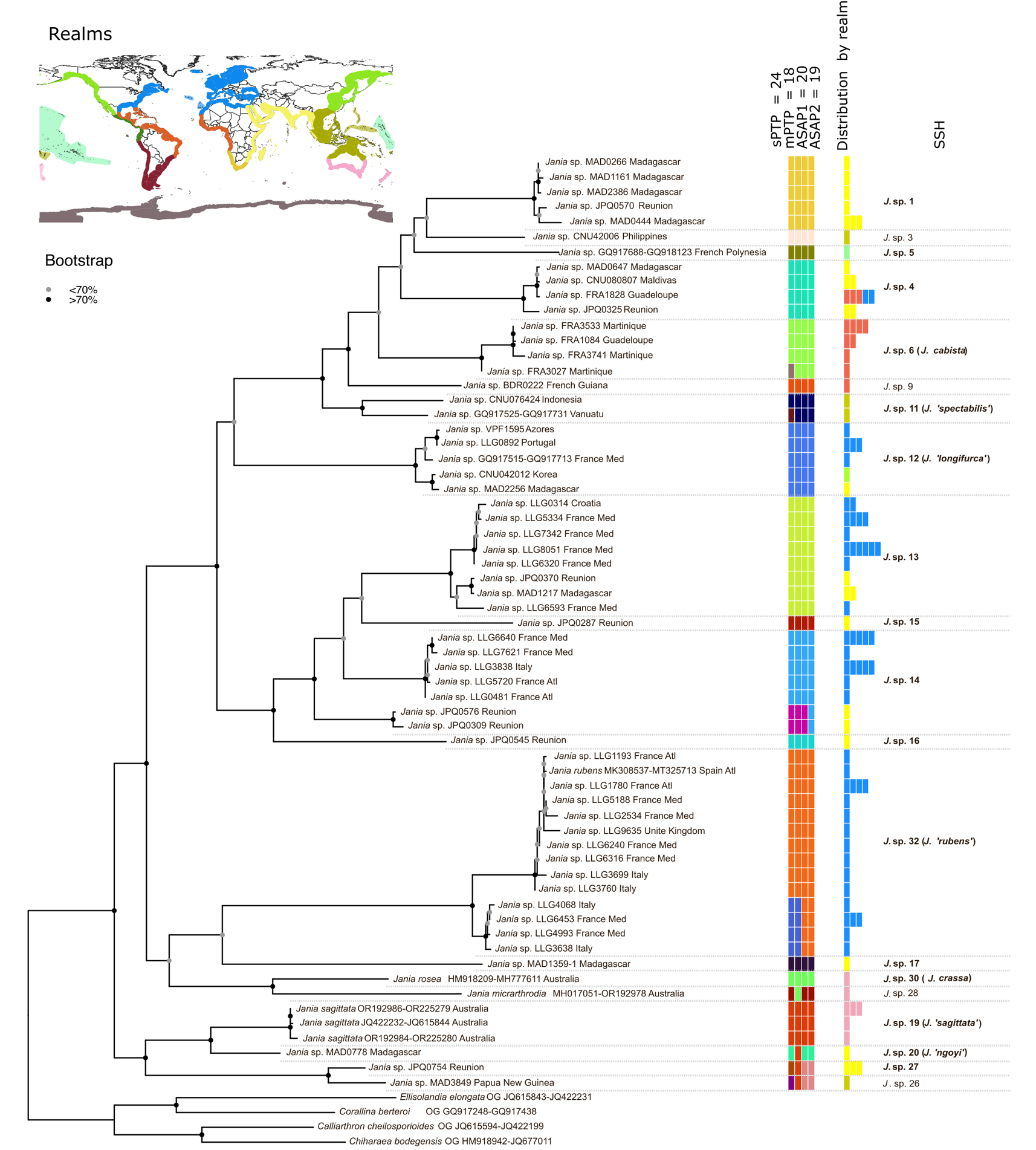


**Figure S2.** Principal Component Analysis of the shape of the intergenicula (n=2 per specimen) for 136 specimens of 21 SSH. The first two principal components accounted for 89.38% of the total variation (PC1 = 78.81%, PC2 = 10.58%). Number of specimens per species: *J. cabista* (N=10), *J. crassa* (N=1), *J. cultrata* (N=2), *J. longifurca* (N=7), *J. ngoyi* (N=1), *J. rubens* (N=21), *J. sagittata* (N=2), *J*. sp. 1 (N=8), J*.* sp. 4 (N=13), *J*. sp. 5 (N=1), *J.* sp. 7 (N=1), *J.* sp. 13 (N=32), *J*. sp. 14 (N=20), *J.* sp. 15 (N=1), *J.* sp. 16 (N=1), J. sp. 17 (N=4), *J.* sp. 23 (N=1), *J*. sp. 24 (N=1), *J.* sp. 27 (N=4), *J.* sp. 35 (N=1), *J. spectabilis* (N=5),


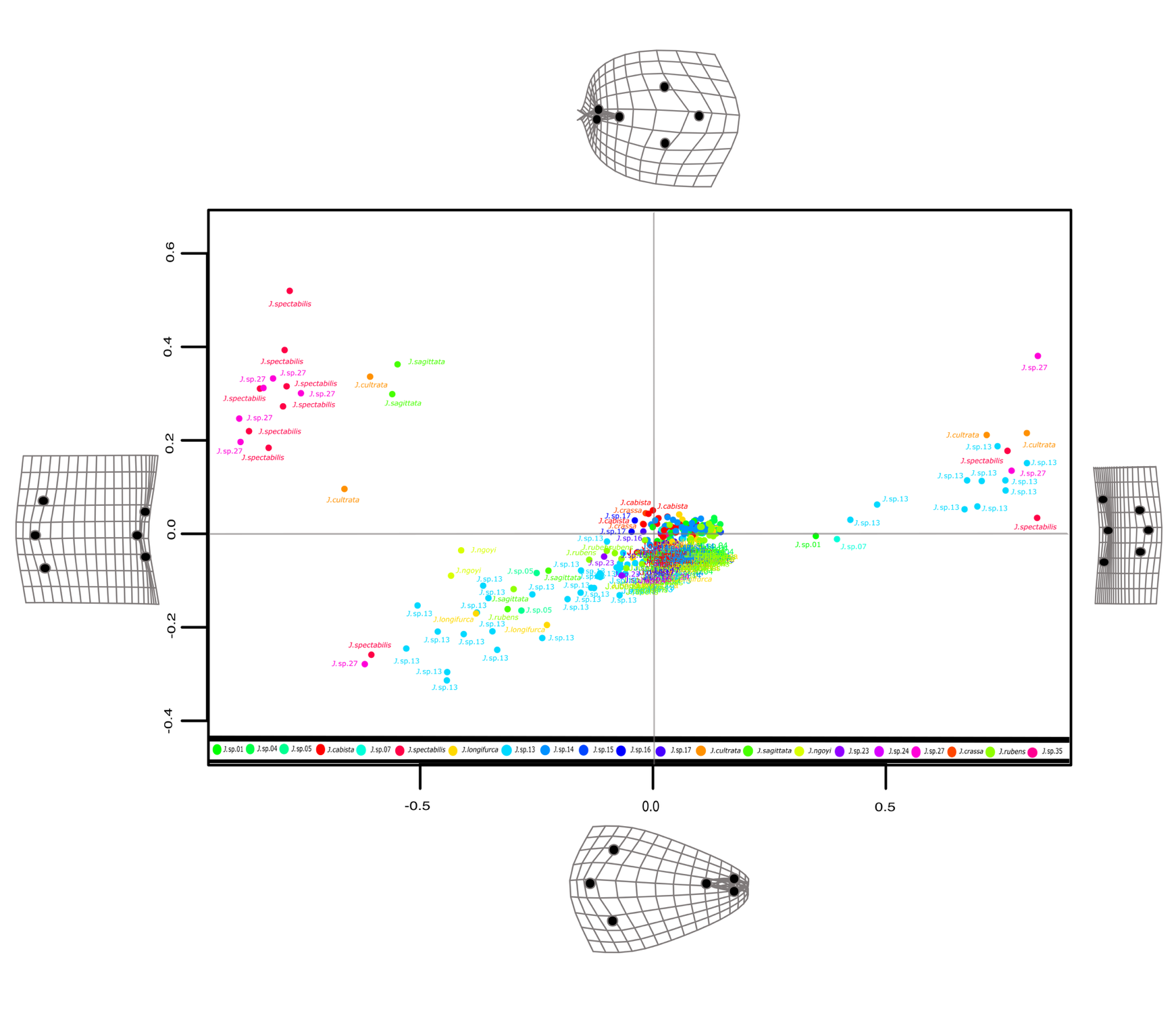


**Figure S3.** Discriminant Analysis (DA) of morphological characters and environmental data for 136 specimens representing 21 SSH. Per specimen, the following mean values were used: intergenicular length (IL, n=10), intergenicular diameter (ID, n=10), angle of ramification (IA, n=10), and mean annual temperature (Tmean). The two discriminant axes explained 85% of the total variation. For putative species with multiple specimens, circles indicate the spread of samples. Sample sizes per species: J. cabista (N=10), J. cultrata (N=2), J. longifurca (N=6), J. ngoyi (N=1), J. rubens (N=19), J. sagittata (N=3), J. sp. 1 (N=8), J. sp. 13 (N=31), J. sp. 14 (N=20), J. sp. 15 (N=1), J. sp. 16 (N=1), J. sp. 17 (N=4), J. sp. 23 (N=1), J. sp. 24(N=1), J. sp. 27 (N=4), J. sp. 4 (N=12), J. sp. 7 (N=1), J. spectabilis (N=4). The biplot shows separation of some SSH, primarily along the first discriminant axis (x-axis). However, many SSH cluster closely, indicating limited discrimination. Intergenicular diameter is strongly correlated with the x-axis, whereas intergenicular length correlates with a diagonal axis running from the top left to the bottom right of the plot.


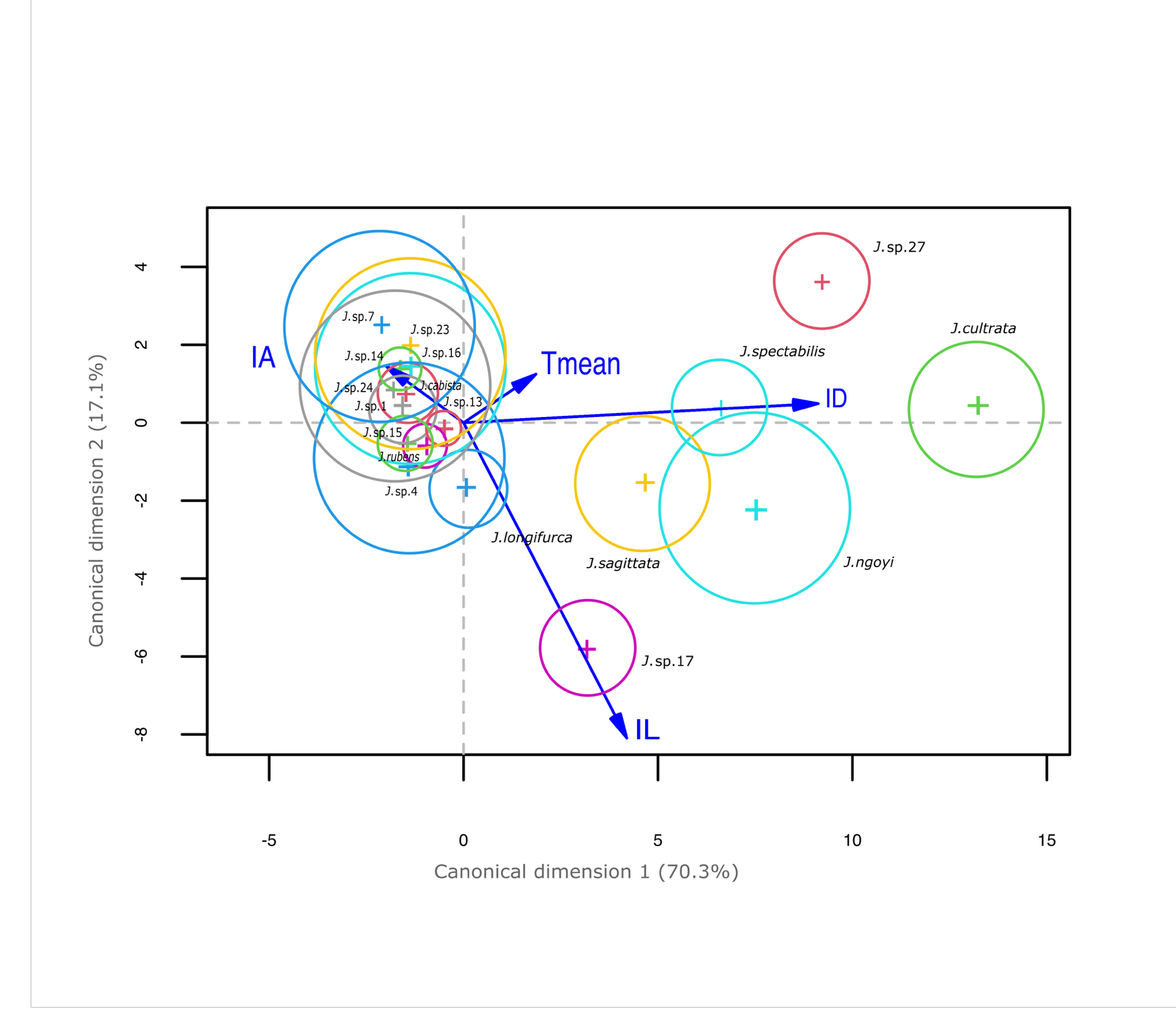


**Figure S4.** Pictures of specimens delineated as *J. ‘rubens’*, the arrow in indicated cornicules. Picture A and B are specimens only from the Mediterranean and pictures C to E are representing specimens from the Mediterranean and the Atlantic A_LLG2729; B_LLG6453; C_LLG3639; D_LLG5198; E_LLG6395.

**
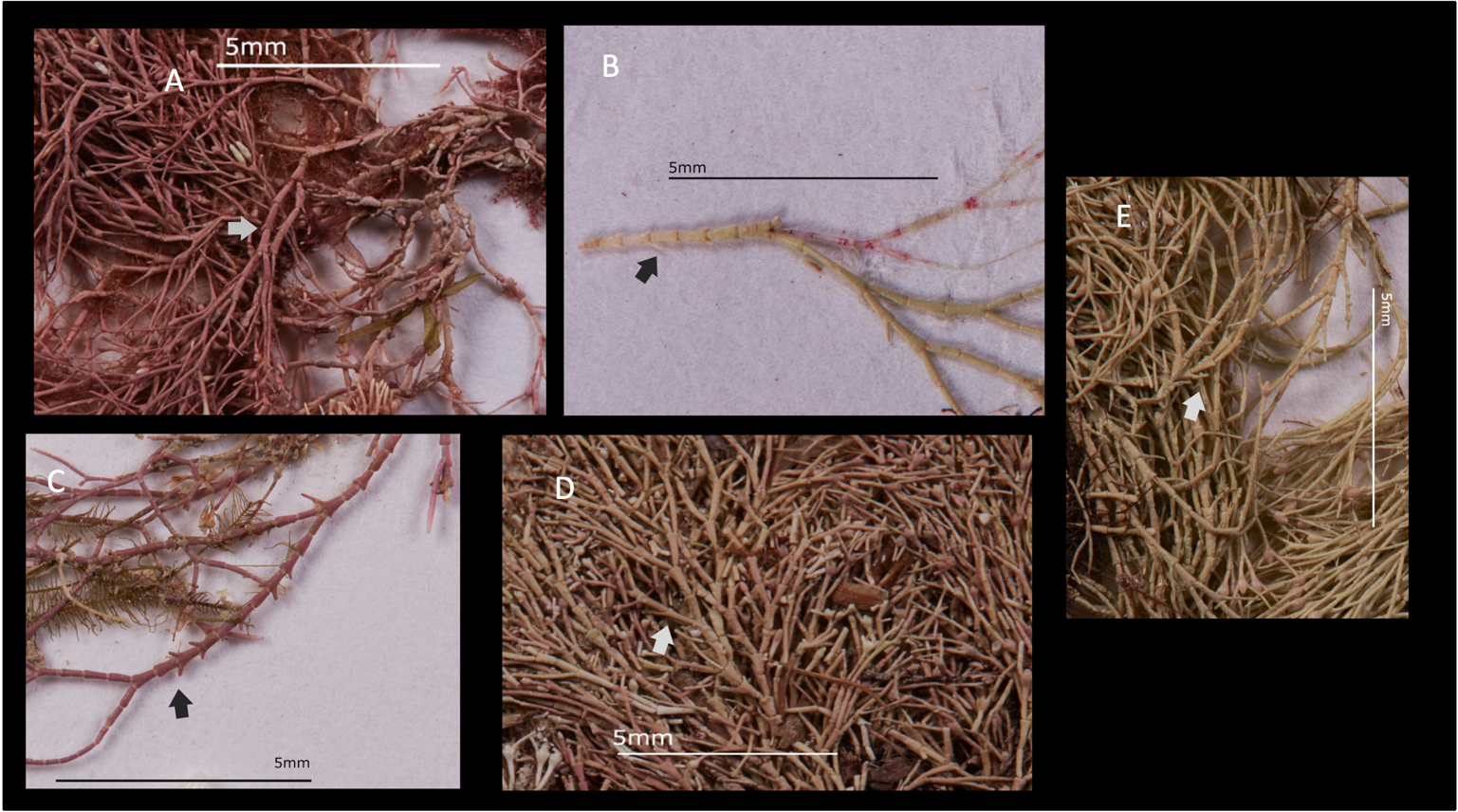
**

**Figure S5.** Pictures of specimens delineated as *J.* sp. 13, the arrow is indicating the pinnate ramification. A_FRA0839; B_ LLG2659; C_LLG6253; D_MAD1365.

**
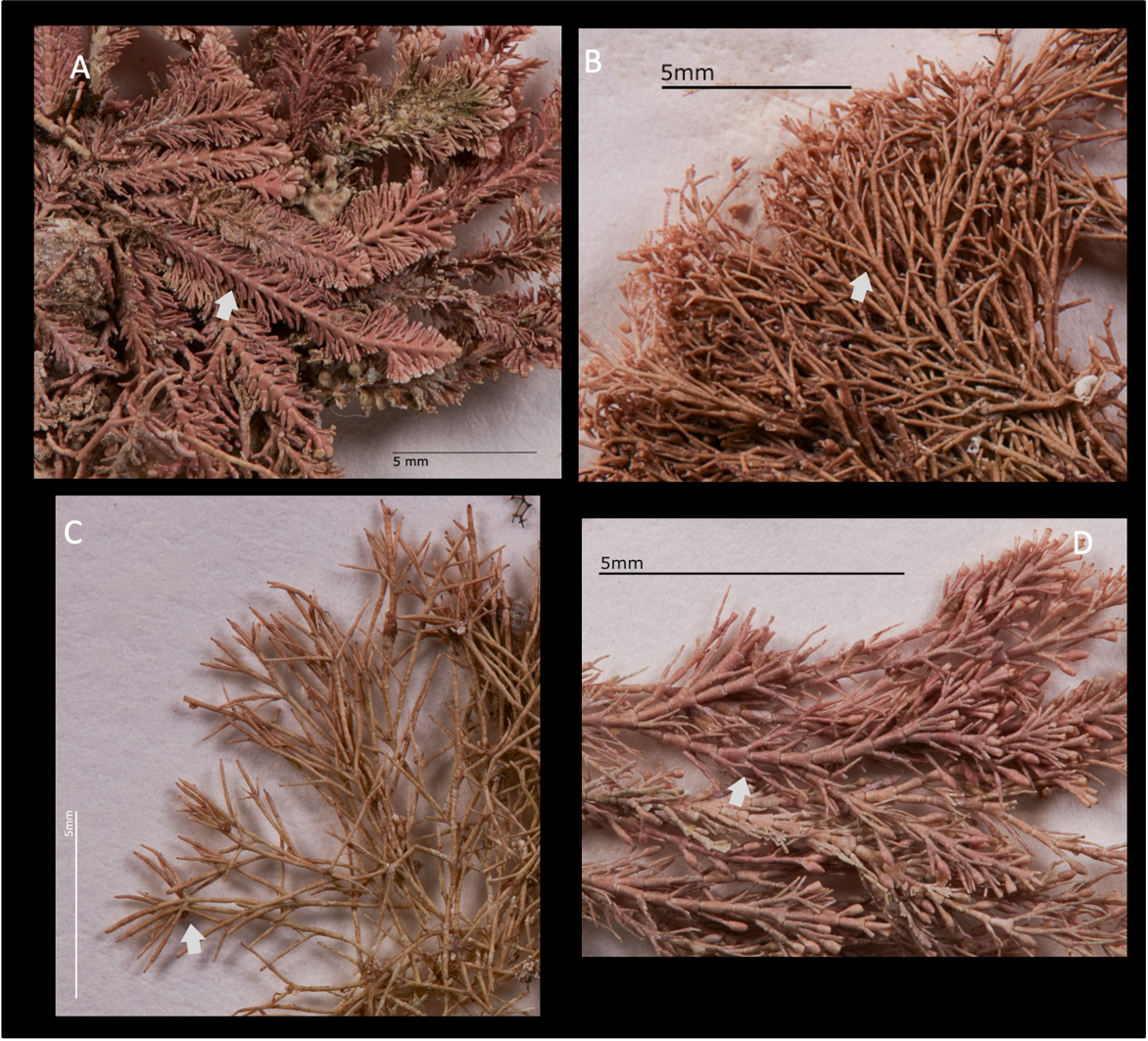
**
